# A cell surface interaction network of neural leucine-rich repeat receptors

**DOI:** 10.1186/gb-2009-10-9-r99

**Published:** 2009-09-18

**Authors:** Christian Söllner, Gavin J Wright

**Affiliations:** 1 Cell Surface Signalling Laboratory, Wellcome Trust Sanger Institute, Hinxton, Cambridge CB10 1HH, UK; 2 Current address: Max Planck Institute for Developmental Biology, Department 3 (Genetics), Spemannstraße 35, 72076 Tübingen, Germany

## Abstract

**Background:**

The vast number of precise intercellular connections within vertebrate nervous systems is only partly explained by the comparatively few known extracellular guidance cues. Large families of neural orphan receptor proteins have been identified and are likely to contribute to these recognition processes but due to the technical difficulty in identifying novel extracellular interactions of membrane-embedded proteins, their ligands remain unknown.

**Results:**

To identify novel neural recognition signals, we performed a large systematic protein interaction screen using an assay capable of detecting low affinity extracellular protein interactions between the ectodomains of 150 zebrafish receptor proteins containing leucine-rich-repeat and/or immunoglobulin superfamily domains. We screened 7,592 interactions to construct a network of 34 cell surface receptor-ligand pairs that included orphan receptor subfamilies such as the Lrrtms, Lrrns and Elfns but also novel ligands for known receptors such as Robos and Unc5b. A quantitative biochemical analysis of a subnetwork involving the Unc5b and three Flrt receptors revealed a surprising quantitative variation in receptor binding strengths. Paired spatiotemporal gene expression patterns revealed dynamic neural receptor recognition maps within the developing nervous system, providing biological support for the network and revealing likely functions.

**Conclusions:**

This integrated interaction and expression network provides a rich source of novel neural recognition pathways and highlights the importance of quantitative systematic extracellular protein interaction screens to mechanistically explain neural wiring patterns.

## Abstract 2

A network of Zebrafish extracellular neuroreceptor interactions are revealed using AVEXIS, a highly stringent interaction assay.

## Background

Identifying the vast number of precise intercellular connections that ultimately account for higher cognitive functions in vertebrate nervous systems, and explaining how they develop, remains one of the main challenges facing neuroscience [1]. Receptor proteins displayed on the surface of neurons are known to relay extracellular recognition events to elicit appropriate cellular responses such as axon guidance, neuron migration and synapse formation, but in comparison to the complex cellular networks that they regulate, relatively few extracellular recognition receptor interactions have been identified [2,3]. Comparative genome analysis and large-scale gene expression studies, however, reveal that vertebrates contain large families of neurally expressed receptor proteins that are expanded relative to invertebrates [4]. These genes are likely to account for the increased complexity of vertebrate nervous systems and two major families are the leucine-rich repeat (LRR) and extracellular immunoglobulin superfamily (IgSF). The neuronal roles of some proteins containing IgSF domains have been well documented (see [5] for a review) but the functions of LRR family members are less well characterized.

**Figure 1:**
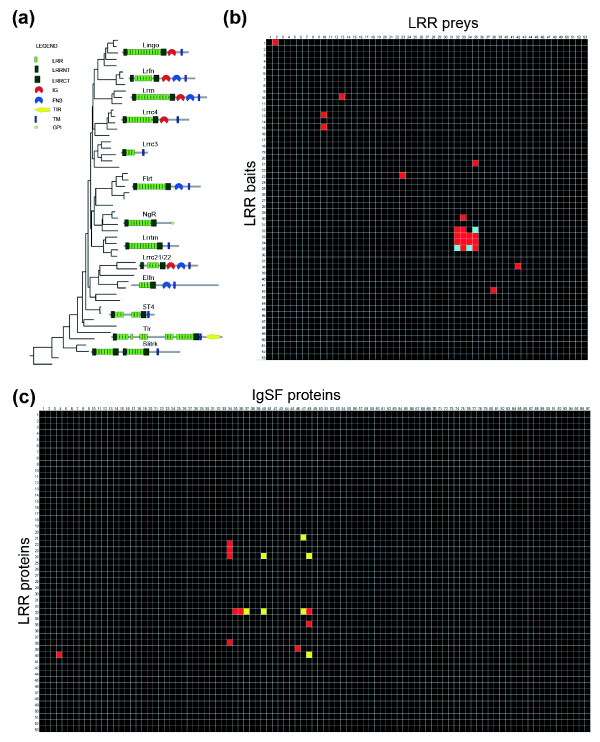
The leucine-rich repeat receptor family and its interactions in zebrafish. **(a) **Zebrafish LRR proteins were phylogenetically clustered into subfamilies using MegAlign (DNASTAR, Madison, WI, USA), and are shown as a phylogenetic tree, together with a schematic representation of their protein architecture. All the proteins shown were included in the protein-protein interaction screen. Protein domain abbreviations: LRR = leucine-rich repeat; LRRNT = leucine-rich repeat amino-terminal domain; LRRCT = leucine-rich repeat carboxy-terminal domain; IG = immunoglobulin superfamily domain; FN = fibronectin type III domain; TIR = Toll/interleukin-1 receptor homology domain; TM = transmembrane region; GPI = glycophosphatidylinositol anchor. **(b) **A binding grid showing all tested reciprocal interactions between the extracellular LRR proteins using AVEXIS. The baits are vertically ordered in correspondence to the tree shown in (a) and numbered as described in Additional data file 4. The preys are similarly ordered horizontally such that homophilic interactions are on the diagonal from top left to bottom right. Interactions identified by a red square were positive in both screens; blue squares were detected only once, but reciprocated. Baits 7, 8, 26 and 50 and prey 43 were expressed below the threshold required for the assay and were therefore not included in the screen. **(c) **A binding grid showing the interaction screen between the zebrafish LRR and IgSF receptor families. The 97 IgSF proteins are ordered horizontally according to their phylogenetic relationships and numbered as described in Additional data file 5; the 52 LRR proteins are similarly arranged vertically. Red and yellow squares indicate high and lower confidence interactions, respectively, as detailed in Additional data file 6.

The cell surface LRR proteins cluster phylogenetically into separate subfamilies with characteristic domain structures (Figure 1a) [6,7]. Even within subfamilies, these genes have discrete and dynamic expression patterns in the developing vertebrate brain and functional analysis also suggests that they have roles in neurodevelopment. For example, genes from the Lrrn subfamily have roles in long-term memory formation [8] and retinal development [9]. Over-expression and/or knockdown of representative members of other subfamilies in neuronal cultures have been shown to have effects on axon outgrowth [10-13], synapse formation [14-16] and axon fasciculation [17]. Nogo receptor 1 (NgR1) and LINGO-1, both members of LRR subfamilies, together with either neurotrophin receptor p75 or TROY, form a receptor complex for myelin components and are responsible for the inhibition of axon regeneration in lesioned mammalian central nervous systems [18]. In addition, genes encoding several LRR proteins have been implicated in neurological disorders, including *LRRTM1 *in schizophrenia [19], *LRRTM3 *in Alzheimer's disease [20], *SLITRK1 *in Tourette's syndrome [21] and *LGI1 *in epilepsy [22].

Despite this involvement in neurological diseases, very little is known about their function and especially their extracellular binding partners. Indeed, of the approximately 20 paralogous subfamilies of membrane-tethered vertebrate LRR-domain-containing receptors [7], extracellular binding partners have been identified for just five: the Lingo, Lrrc4, Flrt, Amigo and NgR subfamilies. One explanation for this disparity is that membrane-embedded receptor proteins are experimentally intractable: they are generally of low abundance and their amphipathic nature makes them difficult to solubilise since they usually contain both large hydrophilic glycans and at least one hydrophobic transmembrane region. Interactions between receptor proteins are also characterised by extremely low interaction strengths, often having half-lives of fractions of a second when measured in their monomeric state [23]. The fleeting nature of these interactions is necessary to permit facile independent motility of migrating cells or growth cones when many receptor proteins arrayed on apposing cell membranes interact. These properties, however, make identifying novel extracellular recognition events mediated through cell surface proteins technically challenging.

The aim of this study was to identify novel receptor interactions that are involved in neural cellular recognition events, focussing in particular on the LRR and also IgSF receptor families. Furthermore, by identifying when and where each gene of an interacting pair is expressed during neural development, we could construct dynamic maps of the neural intercellular recognition program. Using a recombinant protein library of 150 neural receptor ectodomains and a highly stringent interaction assay suitable to detect low affinity extracellular interactions, we identified extracellular binding partners for orphan receptor families - such as the Lrrtms, Lrrns and Elfns - and novel partners for well-characterised receptors, including Unc5b. Paired spatiotemporal gene expression patterns of all genes within the network revealed when and where these interactions might occur during neural development. This neuroreceptor interaction network with integrated gene expression data provides a useful resource to mechanistically explain how complex cellular neural networks develop.

## Results

### A protein interaction network of leucine-rich repeat neuroreceptors

To identify extracellular receptor interactions involved in neural recognition processes, we initially focused on the zebrafish LRR family since they represent a large group of receptor proteins expressed in the nervous system, many of which are 'orphan' receptors. We first identified members of this family by performing a comprehensive bioinformatics search of the zebrafish genome. Orthologues for each of the known type I membrane-tethered and glycophosphatidylinositol-linked mammalian subfamilies [7] were identified and at least one representative was successfully cloned by RT-PCR, with the one exception of the Lrig subfamily (Figure 1a; see Additional data file 4 for a comprehensive list). In total, ectodomain expression constructs were made for 53 genes, which accounts for the vast majority (approximately 80%) of this class of LRR neuroreceptors in the zebrafish genome. To identify novel interactions, we used the AVEXIS (for AVidity-based EXtracellular Interaction Screen) assay developed in our laboratory, which is able to detect very low affinity extracellular interactions (t_1/2 _≤ 0.1 s) and can be scaled to screen thousands of binding events with a very low false positive rate [24]. This assay requires that each ectodomain is expressed as a monomeric biotinylated bait as well as a multimerized, enzyme-tagged prey (Additional data file 1). In total, 49 baits and 52 preys were expressed at sufficient levels and were then normalized prior to screening [24]. The biotinylated monomers were arrayed onto streptavidin-coated microtitre plates, and binary interactions identified by probing these arrays with the prey ectodomains. A primary screen between the LRR receptors of 49 × 52 = 2,548 interaction tests was performed and all positive interactions were then re-tested in both bait-prey orientations in a validation screen using fresh protein preparations. Seventeen interactions between 12 proteins were identified and classified into two confidence categories (Figure 1b; see Materials and methods for full details).

**Figure 2:**
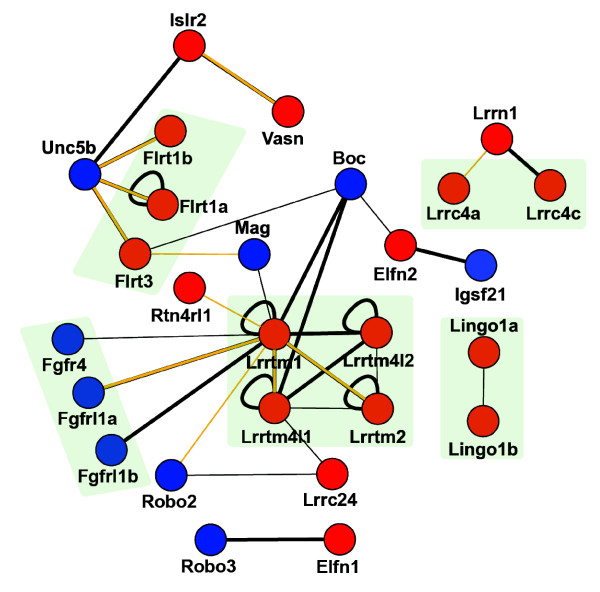
The extracellular LRR and IgSF neuroreceptor interaction network. Receptors belonging to the same paralogous subfamily are grouped and shaded within the network, and interactions classified according to confidence: thick lines = interaction detected in the primary screen and independent of bait/prey orientation; thin line = other detected interactions, including those that are orientation dependent - see Materials and methods and Additional data file 6 for full details. An orange line indicates that the interactions were validated using an independent technique, either surface plasmon resonance (Figure 3) or a bead-binding assay (Additional data file 2). IgSF-receptors = blue nodes, LRR-receptors = red nodes.

Essentially all of the interactions in the LRR neuroreceptor network (Figure 2) were novel, with only the homophilic Flrt1a interaction having been previously described [25]. The network contained the first reported extracellular interactions for the Lrrtm and Lrrn orphan receptor subfamilies. Interacting receptor pairs were often found to involve several members of a subfamily, suggesting that the interacting binding face is conserved between related proteins. For example, all four Lrrtm subfamily members were able to form both homo- and heterotypic interactions between themselves, and Lrrn1 interacted with two out of four members of the Netrin-G1 ligand/Lrrc4 subfamily [14]. We also identified interactions between LRR proteins, which could not be clustered into subfamilies such as the Islr2-Vasn interaction.

### LRR neuroreceptors have binding partners within the IgSF

Since the IgSF is a well documented receptor family for LRR domains [26,27], we next systematically screened the LRR proteins against a large library of 97 bait ectodomains belonging to the zebrafish IgSF (see Additional data file 5 for a comprehensive list). In total, 52 × 97 = 5,044 interactions were screened and positive interactions were subsequently retested using independent protein preparations in both bait-prey orientations. A further 17 interactions involving nine IgSF proteins were added to our neuroreceptor interaction network and similarly placed into two confidence categories (Figure 1c).

All interactions within the LRR-IgSF network except one [28] were previously unknown. The systematic nature of the screen revealed novel extracellular interactions for well described axon guidance receptors. For example, we identified novel LRR-domain-containing transmembrane ligands for the receptors Robo2 and 3, which we have shown bind to zebrafish Slit proteins (see Materials and methods) demonstrating that they were functionally active. Robo2 interacted with Lrrc24 and Lrrtm1, and Robo3 with Elfn1, suggesting that the Robo receptors are able to respond to local membrane-tethered signals in addition to secreted ligands such as Slit. Similarly, Unc5b, a known receptor for Netrin [29,30], interacted with three out of the four Flrt-family homologs [31] (Figure 2). Other IgSF-LRR receptor interactions were found for the Lrrtm1 protein, which interacted with three out of four fibroblast growth factor receptor homologs in the screen (Fgfr4, Fgfrl1a and Fgfrl1b), and novel binding partners for both the axon guidance receptor Boc, and the myelin-associated glycoprotein Mag.

### Interaction strengths between related neuroreceptors quantitatively vary

**Figure 3:**
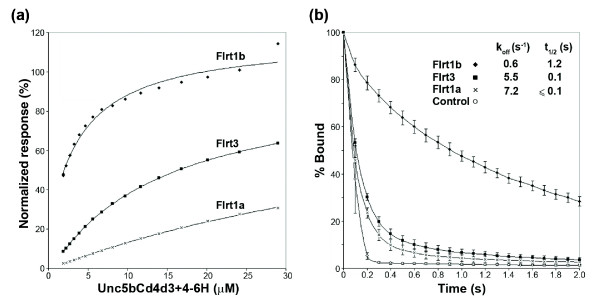
Interaction strengths between Unc5b and Flrt paralogs are surprisingly heterogeneous. **(a) **Equilibrium binding analysis of Unc5b and three Flrt paralogs. Different concentrations of purified, monomeric Unc5b-Cd4d3+4-6H were injected over streptavidin-coated flow cells upon which biotinylated baits - Flrt1a (1018 RU), Flrt1b (984 RU), Flrt3 (1027 RU) - and control Cd4d3+4 were immobilized. The amount of bound Unc5b was calculated by subtracting responses in the control flow cells from those in the Flrt-immobilized cells once equilibrium had been reached. Equilibrium dissociation constants (K_D_s) were obtained by fitting a non-linear binding curve to the data. To facilitate comparison, the binding responses were normalized by using the predicted R_max _from the fit to the data. **(b) **Kinetic analysis of the Unc5b-Flrt interactions. Off-rate constants (k_off_) were calculated by globally fitting a first order decay curve to the dissociation phase of three concentrations of Unc5b; half-lives (t_1/2_) were calculated as t_1/2 _= ln2/k_off_. Shown are the normalized, averaged values (error bars = ± 1 standard deviation, n = 3). On-rate constants (k_on_) were calculated in the same way using an association model and were > 1 × 10^5 ^M^-1^s^-1 ^in all cases.

One notable feature of our network is that nearly half (48%) of the receptors have more than one heterophilic binding partner (Figure 2). In all cases, each receptor combination had compatible expression patterns, with its multiple binding partners expressed in overlapping territories (see below), raising the possibility of binding competition at the cell surface. In an attempt to resolve this problem, we asked to what extent the strengths of interactions between shared receptors might vary. We selected a subnetwork of interactions involving Unc5b and the Flrt paralogs and determined their relative interaction strengths using monomeric proteins and surface plasmon resonance. The ectodomain of Unc5b was expressed as a secreted Cd4d3+4-6His-tagged protein using mammalian cells, purified and eluted as a monodisperse peak using gel filtration (data not shown). The equilibrium dissociation constant (K_D_) was calculated by injecting dilutions of monomeric purified Unc5b over each of the three biotinylated Flrt baits immobilized on a streptavidin-coated sensor chip; the reference-subtracted binding responses at equilibrium were plotted against the injected Unc5b concentration (Figure 3a). As expected, the K_D_s were in the micromolar range, but varied considerably from the relatively strong approximately 4 μM (Flrt1b) interaction where saturable binding was evident, through approximately 14 μM (Flrt3) to the very weak Flrt1a interaction, the K_D _of which could not be estimated reliably using the injected concentrations of Unc5b protein, but was in excess of 50 μM. A kinetic analysis of the interactions was consistent with the equilibrium binding data, with off rate constants (k_off_) varying from 0.6 s^-1 ^(t_1/2 _= 1.2 s) for Flrt1b to ≥ 7.0 s^-1 ^(t_1/2 _≤ 0.1 s) for Flrt1a (Figure 3b). These measurements show that interactions between neuroreceptors within our network have a low affinity and vary considerably in their binding strength, even between proteins belonging to the same paralogous family.

### Paired receptor gene expression patterns reveal dynamic cellular neural recognition maps

The binding network of IgSF and LRR receptors (Figure 2) is a static representation of possible extracellular protein interactions and does not reflect the spatial and temporal ordering of recognition events used in the developing nervous system. To reveal when and where these binding events might occur, we determined the expression patterns of all the receptor genes within the network at four stages of zebrafish embryonic development (Additional data file 7 and see Materials and methods for details of an online database of paired stage and orientation-matched images) using mostly two-color fluorescent *in situ *hybridization to directly compare the expression of each gene encoding an interacting receptor pair within the same embryo.

**Figure 4:**
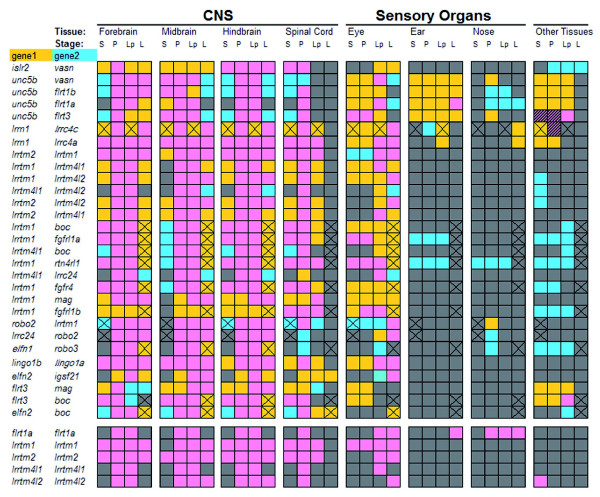
Genes encoding interacting receptors show compatible spatiotemporal expression. Genes encoding interacting receptors are paired (gene 1, gene 2) and listed vertically; homophilic interactions were treated separately below. The expression in anatomically distinct regions of the nervous system at different stages of embryonic development is indicated by appropriate shading within the grid. Expression key: gene 1 only = gold; gene 2 only = blue; co-expression = pink; no expression = grey; hatched = both expressed at the same stage outside the nervous system but not in identical or neighboring tissues; cross = expression not determined for one of the two genes. Stages: S = 14 to 19 somites; P = Prim5; Lp = Long-pec; L = Larval (4 to 5 days post-fertilization). CNS = central nervous system.

The expression pattern of each gene encoding an interacting pair was summarized by plotting a grid of time-resolved tissues within the central and peripheral nervous systems, highlighting where each pair was spatiotemporally congruous (Figure 4). All heterophilic receptor pairs had compatible local tissue expression, usually at several different stages of development, providing independent biological support for the interaction network. All interacting receptor pairs were compatibly expressed within the central nervous system between the 24 and 48 hours post-fertilization stages, coinciding with an active period of neural development, including neuron migration and pioneering axonal outgrowth. In contrast, fewer of the interacting pairs were compatibly expressed in the sensory systems, such as the retina, and especially the acoustic and olfactory systems. Several receptors were also expressed in other tissues, although these were, in general, not spatially compatible with their binding partners in the network. This suggests the existence of additional binding partners for these receptors outside of the developing nervous system.

**Figure 5:**
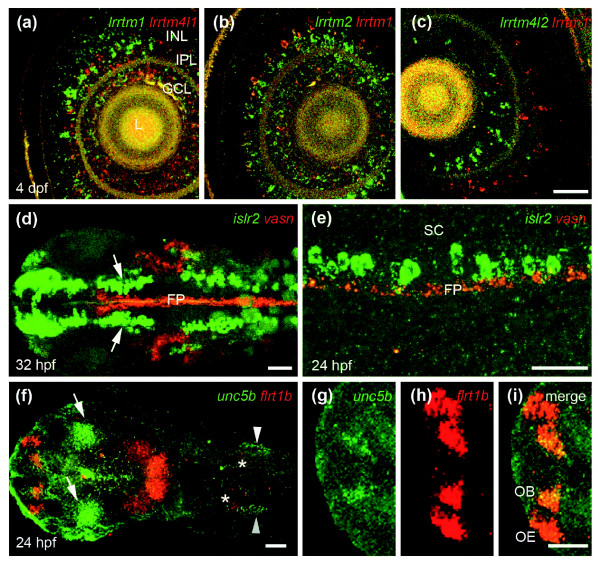
Two-color wholemount *in situ *hybridization of interacting neuroreceptors. **(a-c) **Single optical sections showing largely non-overlapping expression of the Lrrtm genes within the inner nuclear and ganglion cell layers of 4 days post-fertilization zebrafish retinae. Note that the confluent yellow staining within the lens represents background auto-fluorescence in both channels.  **(d-e) **Neuron-glia interactions. (d) Dorsal view of the head region of a 32 hour post-fertilization (hpf) zebrafish embryo: *vasn *(red) is expressed in the most ventral part of the spinal cord in the medial floor plate cells (FP). *islr2 *(green) is expressed in fore-, mid- and hindbrain neurons; note that the midbrain neurons are in direct contact with the floor plate (arrows). (e) Lateral view of the developing spinal cord of a 24 hpf zebrafish embryo showing discrete cells within the spinal cord (SC) that are directly adjacent but dorsal to the floor plate. **(f-i) **Dorsal views of a 24 hpf zebrafish embryo showing expression of *unc5b *(green) and its interacting partner *flrt1b *(red). (f) *unc5b *is expressed in the dorsal retina (arrows) and the ear (arrowheads), *flrt1b *in the dorsal regions of the lateral midbrain and mid-hindbrain boundary; expression is also detectable in the vestibulo-acoustic ganglion (asterisks). (g-i) Higher magnification of the forebrain showing that *unc5b *is also expressed in the medial part of the olfactory bulb (g) where it overlaps with the *flrt1b *staining (h) in the olfactory bulb (OB) and olfactory epithelium (OE) (i). Scale bars: 50 μm (a-c); 80 μm (d); 40 μm (e); and 50 μm (f-i). GCL = retinal ganglion cell layer; INL = inner nuclear layer; IPL = inner plexiform layer;  L = lens; OB = olfactory bulb; OE = olfactory epithelium.

While such a summary provides a useful low resolution overview, further functional insights can be gained by correlating the detailed expression patterns of interacting receptors to known neurobiologies: we provide three examples. Firstly, the Lrrtm family of receptors - which all interacted with each other - showed a complex pattern within the developing brain (Additional data file 7) but, most remarkably, were also expressed in a largely mutually exclusive pattern within the retina (Figure 5a-c). This receptor family could therefore be a source of intercellular molecular recognition cues required for directing the connectivity of the many cellular subtypes within the retina [32]. In our second example, the gene encoding the Vasn protein was expressed in the specialized glial cells that make up the floor plate of the spinal cord (Figure 5d, e). Its receptor, encoded by the *islr2 *gene, was expressed by head and spinal neurons (Figure 5d, e), including motoneurons whose axons are known to directly contact the floor plate as they innervate ipsilateral muscle fields [33]. In our last example, the expression patterns of the genes encoding the interacting receptors Flrt1b and Unc5b were consistent with a role in regulating retinotectal mapping: *unc5b *was restricted to the dorsal region of the developing retina from mid-somitogenesis stages whereas its binding partner, *flrt1b*, was expressed in the tectum (Figure 5f). Unc5b-Flrt1b and Flrt1b homophilic interactions could also be involved in neural recognition within the olfactory system since *flrt1b *was expressed in the olfactory epithelium, and *unc5b *and *flrt1b *were co-expressed in the olfactory bulb at 24 hours post-fertilization (Figure 5g-i). Overall, we frequently observed overlapping or directly abutting expression for interacting neuroreceptors within the developing nervous system (for examples, see Additional data file 3). Therefore, in addition to providing starting points to identify novel signaling pathways for known neurobiologies, the receptor interaction network coupled with the developmental gene expression patterns is a useful resource to also identify new potential cellular interactions on the basis that they compatibly express interacting receptors.

## Discussion

This study represents the first step towards mapping an extracellular interaction network between neural receptor proteins, a resource that will be necessary to understand the intercellular recognition processes that ultimately underlie brain development and function. The importance of understanding these processes is becoming increasingly apparent as neurological disorders are more frequently being viewed as a product of abnormal brain development [34]. Significantly, we have described here binding partners for three orphan LRR receptor subfamilies, including the Lrrtms, which have been implicated in neurological diseases, including schizophrenia. While the LRR and IgSF are both large families of neurally expressed receptors, there are several other families of cell surface proteins that contribute to neural recognition processes. A comprehensive extracellular network of interactions within the developing nervous system will require the addition of these protein families to our protein library. Crucially, however, we have shown that the systematic screening approach using the AVEXIS method has the scalability and sensitivity to detect transient interactions that are not generally detected by other high throughput protein binding assays. Beyond identifying extracellular binding partners for orphan receptor families, this systematic unbiased method can identify additional binding partners for receptors that already have identified ligands.

Currently, our protein library contains approximately 80% of the zebrafish neural LRR receptors, providing a high density coverage for this family of receptors, which are known to be important for synaptic target selection [35]. We have shown that LRR receptor proteins are able to form both homophilic and heterophilic interactions within the family but also interact with receptors from the IgSF. Despite this large scale approach, we did not identify binding partners for all LRR subfamilies; indeed, both the Slitrk and Lrrc3 subfamilies still have no documented extracellular binding partner. LRR receptors are also known to bind other protein families such as the Netrin-G [14] and tumor necrosis factor-receptor family [36] and the future inclusion of these receptor families into our interaction screens is likely to reveal further binding partners for these subfamilies.

The AVEXIS assay was developed and implemented at a high stringency threshold to effectively eliminate false positives so as to produce high-quality datasets [24]. Using this stringency, approximately 0.5% of unique interactions screened - calculated using just one bait-prey orientation - are positive. Although difficult to directly compare due to the ascertainment biases inherent in selecting proteins restricted to a particular subcellular localization (such as the plasma membrane) or screening within protein families previously demonstrated to interact, this interaction frequency lies between large-scale binary yeast-two-hybrid assays (approximately 0.01%) [37] and the LUMIER assay (approximately 8%) [38]. The paucity of zebrafish protein interaction data makes a false negative rate difficult to assess, but by using the closest mammalian orthologue, the main class of false negatives comprised homophilic interactions. This is most likely due to prey-prey associations [24], although it should be noted that AVEXIS is able to detect some homophilic interactions and further work is required to determine the biochemical and/or structural reasons for this difference. A complementary scalable assay dedicated to identifying homophilic receptor interactions has been developed and could be used to specifically detect this class of interactions [39]. AVEXIS may also not be generally suitable to detect interactions between ectodomains that interact in *cis *to form co-receptor complexes since no interaction between NgR1 and Lingo1 ectodomains was detected [40]. During the preparation of this manuscript, an independent study reported the Flrt3-Unc5b interaction in *Xenopus *and demonstrated its role in cell adhesion processes during early embryogenesis [28].

The systematic nature of our screening approach revealed that many receptors have multiple binding partners with compatible expression patterns, raising the possibility of binding competition at the cell surface. While parameters such as abundance, local clustering and accessibility will also influence binding *in vivo*, the intrinsic binding affinity of a ligand for its receptor is important for resolving and measuring these effects. The finding that the three Flrt paralogs have different binding affinities for the Unc5b receptor, spanning at least an order of magnitude, was surprising and is likely to influence their ability to initiate signaling *in vivo*. Quantitative measurements of adhesion receptors in the immune system have shown that solution interaction strengths weaker than approximately 50 μM are unlikely to be high enough to support spontaneous interactions at physiological surface densities, highlighting the functional relevance of these measurements [41].

## Conclusions

We have initially focused on two large families of neural receptor proteins - LRR and IgSF - as a starting point to begin a systematic approach to identify all extracellular recognition events required in the development of the vertebrate nervous system. In principle, this approach could be applied to other receptor families and secreted ligands. We anticipate that these networks of recognition receptors interpreted in the context of their corresponding gene expression patterns will provide a valuable new resource for neurobiology and will stimulate further research into the functional role of these interactions.

## Materials and methods

### Zebrafish husbandry

Zebrafish were maintained on a 14/10 hour light/dark cycle at 28.5°C according to UK Home Office and local institutional regulations, and staged according to Kimmel [42]. Embryos used for *in situ *hybridization were the progeny of a WIK/*alb *outcross; *alb/alb *embryos were used where endogenous pigment obscured staining signals.

### Gene cloning and ectodomain library construction

The entire predicted extracellular and transmembrane regions of cell surface LRR-domain-containing genes were amplified by RT-PCR from mixed-stage zebrafish cDNA using oligonucleotides designed from automated gene predictions of the zebrafish genome [43]. PCR products were either cloned or used as further templates to amplify predicted ectodomains, which were ligated into a mammalian expression vector based on pTT3 [44]. The protein library was produced as previously described [24].

### Interaction screen

Interactions were identified using the AVEXIS procedure as described [24]. Each plate contained both negative and positive controls as shown in Additional data file 1. Negative controls were the plate prey presented to the baits rat Cd4d3+4 (well H7), Cd200R (H8) and Cd200 (H9). Positive controls were Cd200R prey and Cd200 bait (H10) and the Cd200 bait diluted 1:10 (H11) and 1:100 (H12). Fifty-two LRR prey proteins were systematically screened against 49 LRR and 97 IgSF ectodomain bait proteins derived from membrane-bound receptors [24]. The vast majority of the LRRs and 28 of the IgSF proteins (indicated in Additional data file 5) were initially screened in both bait-prey orientations. Protein pairs that showed positive interactions in the first-pass screen were re-expressed and systematically re-screened in the same matrix-style manner as both baits and preys in an independent validation screen. Interactions that were positive in the first screen and could be detected in a reciprocal fashion were considered as high confidence interactions. Other interactions, such as those that were dependent upon the bait-prey orientation, were regarded as lower confidence interactions. Full details of the screening results are shown in Additional data file 6 and the protein interactions from this publication have been submitted to the International Molecular Exchange Consortium (IMEx) [45] through IntAct (pmid: 17145710) and assigned the identifier IM-11659. Expected interactions, including those between the zebrafish Robo and Slit orthologs, were detected in subsequent and ongoing interaction screens showing that the recombinant proteins are functionally active and full details are available at IntAct: Robo1-Slit1b, 2, 3 (EBI-2268920, EBI-2269164, EBI-2269173), Robo2-Slit2 (EBI-2269141) and Robo3-Slit1b, 2 (EBI-2269026, EBI-2268001).

### Fluorescent bead binding

The extracellular regions of rat Cd200, Lrrn1, Vasn, Robo2, Lrrtm1, Unc5b and Flrt3 used in the AVEXIS screening were cloned into a pTT3-based expression vector to produce a chimeric construct that contained the transmembrane domains of the rat Cd200R and the green fluorescent protein in the cytoplasmic region. HEK293E cells were transfected with these constructs, harvested 2 to 3 days later, washed three times in phosphate-buffered saline/1% bovine serum albumin, vortexed and approximately 5 × 10^5 ^cells aliquoted into each well of a flat-bottomed 96-well microtitre plate. Interactions were then detected using a modified version of the fluorescent bead binding experiments described in [46]. Cells were then presented to biotinylated bait proteins immobilised around streptavidin-coated Nile Red fluorescent 0.4 to 0.6 μm microbeads (Spherotech Inc., Lake Forest, IL, USA) at a ratio of approximately 120 beads per cell. After incubating for an hour on ice the cells and beads were resuspended in 250 μl of phosphate-buffered saline/1% bovine serum albumin and analyzed for binding events using a BD LSR II flow cytometer and the data were analysed using FlowJo v7.5.3 software (Tree Star, Inc., Ashland, OR, USA).

### Protein purification and BIAcore analysis

Protein purification and BIAcore analysis were performed as described [24]. Briefly, the ectodomain of Unc5b was produced in mammalian cells as a Cd4d3+4-6His-tagged protein and purified on a 1 ml His-Trap column (GE Healthcare, Amersham, Bucks, UK). Protein aggregates, which are known to influence kinetic experiments, were removed by gel filtration using a 125 ml Superose6 column prior to BIAcore analysis. The indicated amounts of the Flrt-Cd4d3+4-bio baits were immobilized onto a streptavidin-coated sensor chip and approximate molar equivalents of Cd4d3+4-bio were used as a reference. All binding studies were performed in HBS-EP buffer (GE Healthcare, Amersham, Bucks, UK) at zebrafish physiological temperature (28°C). Flow rates of 100 μl min^-1 ^were used for kinetic studies to minimize rebinding effects and data were collected at the maximum rate of 10 Hz. Equilibrium dissociation and both on and off rate constants were calculated using the appropriate fitting model in the BIAevaluation software.

### *In situ *hybridization

Fluorescent two-color wholemount *in situ *hybridizations were essentially carried out as described [47]. RNA probes were prepared from a template amplified from the protein expression constructs encoding the entire ectodomain fragments. To facilitate comparison, single color images of the gene expression patterns at several stages of development were stage and orientation-matched and are presented in an online database at [48]. Expression data are also publicly available at [49].

### Microscopy

Fluorescently labeled zebrafish embryos were mounted in Vectashield mounting medium (Vector Laboratories, Burlingame, CA, USA) and images were captured either on a Leica SP5 confocal microscope or a Zeiss Axioplan2 compound microscope fitted with a Volocity OptiGrid structured light device (Improvision, Coventry, UK).

## Abbreviations

AVEXIS: avidity-based extracellular interaction screen; IgSF: immunoglobulin superfamily; LRR: leucine-rich repeat.

## Authors' contributions

CS performed all experiments and prepared the figures except for the BIAcore analysis, which was done by GW. The manuscript was written by GW.

## Additional data files

The following additional data are available with the online version of this paper: a figure showing an outline of the AVEXIS procedure (Additional data file 1); a figure showing validation of interactions using a fluorescent bead-based assay (Additional data file 2); a figure showing that interacting neuroreceptors display both complementary and overlapping expression patterns in the developing brain (Additional data file 3); a table listing the zebrafish LRR genes cloned and used to produce recombinant ectodomains (Additional data file 4); a table listing the 97 zebrafish IgSF ectodomain baits (Additional data file 5); a table classifying the neuroreceptor interactions using AVEXIS (Additional data file 6); a table listing the spatiotemporal expression of each gene within the interaction network (Additional data file 7).

## Supplementary Material

Additional data file 1 **(a) **The entire ectodomain of each LRR receptor was expressed in mammalian cells as both a bait and a prey. The bait proteins were biotinylated monomers, each containing a carboxy-terminal tag of the rat CD4 domains 3 and 4 and an enzymatically biotinylatable sequence. The prey also contained the rat CD4 tag but was followed by a pentamerization sequence derived from rat cartilage oligomeric matrix protein (5°) and the beta-lactamase enzyme. The expression levels of both bait and prey were measured and normalized. **(b) **The library of bait proteins was arrayed on a streptavidin-coated 96-well microtiter plate and a normalized prey protein added. After a brief wash, binding was determined by adding the colorimetric beta-lactamase substrate, nitrocefin: positive wells turned red. **(c) **Actual screening plates showing the Islr2/Slit-like2 interaction detected in a reciprocal fashion. The left panel shows Islr2 as the bait protein and Slit-like2 as the prey; the right panel shows the interaction in the reciprocal orientation. Click here for file

Additional data file 2 Interactions identified using AVEXIS were validated by immobilizing the biotinylated bait protein around a Nile Red fluorescent bead (shown on the y-axis) and presenting them to cells transfected with the extracellular regions of cell surface receptor proteins expressed as a TM-GFP chimera (x-axis). The percentage of events counted in each of the quadrants (Q1 to Q4) is shown. **(a,b) **Controls showing positive staining of rat Cd200-TM-GFP with rat Cd200R-coated beads (a) but not Cd4d3+4-coated beads (b). **(c-f) **Examples of interactions Lrrc4a-Lrrn1 (c), Islr2-Vasn (d), Lrrtm1-Robo2 (e) and Mag-Flrt3 (f) showing beads associating with transfected cells. Interactions were called as positive when beads preferentially associated with GFP-positive transfected cells (Q2:Q1 was greater than 1). Click here for file

Additional data file 3 **(a-c) **Dorsal view of the zebrafish midbrain at 24 hours post-fertilization showing the *nlrr1 *gene (a) is expressed throughout the neuroepithelium of the midbrain, whereas its receptor, *ngl2 *(b), is restricted to two lateral midbrain domains that directly abut the *nlrr1 *expression domain (c). **(d-f) **A single optical section through the brain of a 4 days post-fertilization (dpf) zebrafish larva showing that *robo2 *(d) and *lrrtm1 *(e) are expressed in restricted patterns within all brain regions. The merge (f), shows largely non-overlapping, adjacent expression, particularly in a forebrain nucleus (arrowheads) and the hindbrain (arrows). **(g-i) **Dorsal view of the forebrain and partial midbrain of a 32 hours post-fertilization zebrafish larva showing that *lingo1a *(g) and *lingo1b *(h) are expressed in partially overlapping domains in the telencephalon (i). **(j) **Lateral view of a 4 dpf zebrafish larva showing *robo3 *expression in green and *elfn1 *expression in red. Both genes are expressed in the habenula nucleus in the dorsal forebrain (arrowhead). **(k-m) **Dorsal view of the forebrain region of a 5 dpf zebrafish larva showing that *robo3 *is expressed symmetrically in both habenula nuclei (k), whereas *elfn1 *(l,m) is expressed asymmetrically with higher expression levels in the left nucleus (lHB). All images are two-color wholemount *in situ *hybridizations with anterior left; cartoons depicting the interacting receptor-ligand pairs are shown in the left panels. Scale bar: 50 μm (a-c,d-f,g-j,k-m); 93 μm (j). Click here for file

Additional data file 4 Each gene is numbered according to its phylogenetic relationship as shown in Figure 1a and therefore clustered into LRR subfamilies as indicated. Listed for each gene are a systematic name with a *cssl:d0 *prefix, the current official ZFIN nomenclature, a proposed new nomenclature where appropriate (and used throughput this paper), GenBank accession number, the final carboxy-terminal amino acid of the ectodomain at which the truncation was made (the truncation site, 'Trunc.') and the closest human BLASTP match together with the percentage sequence identity. Click here for file

Additional data file 5 Each IgSF ectodomain is numbered in the order of its phylogenetic relationship and corresponds to the numbers in the LRR-IgSF binding grid (Figure 1c). Each gene is given a systematic identifier, the cssl:d prefix, which is listed together with the current official ZFIN nomenclature (note identical gene names indicate splice variants), GenBank accession number and the closest human BLASP match, together with the percentage sequence identity. Twenty-eight proteins indicated by asterisks were also produced as prey proteins and, therefore, screened in both bait-prey orientations. One protein, Sc:d805 (number 28 in the table), interacted with > 50% of the library as both a prey and a bait and was therefore excluded from subsequent analysis. Click here for file

Additional data file 6 Interactions were classified into 12 groups (A to L) according to their behavior in the interaction screen using AVEXIS as taken from [24]; according to this scheme, no class B interactions were categorized. Interactions were considered as high confidence if they were positive in the primary screen and could be detected in both bait-prey orientations in either the primary or validation screens (classes A, C, D, E, F). IntAct accession numbers for both bait-prey orientations are provided where applicable. Click here for file

Additional data file 7 The expression pattern of each neuroreceptor was determined by wholemount *in situ *hybridization at the indicated stages during zebrafish development. Images are freely available at [49]or integrated with the interaction network at [48]. Click here for file
