# Supplementary figures and images for: A cell surface interaction network of neural leucine-rich repeat receptors
Source: Genome Biol. 2009 Sep 18;10(9):R99. doi: 10.1186/gb-2009-10-9-r99 (PMC2768988; doi:10.1186/gb-2009-10-9-r99)

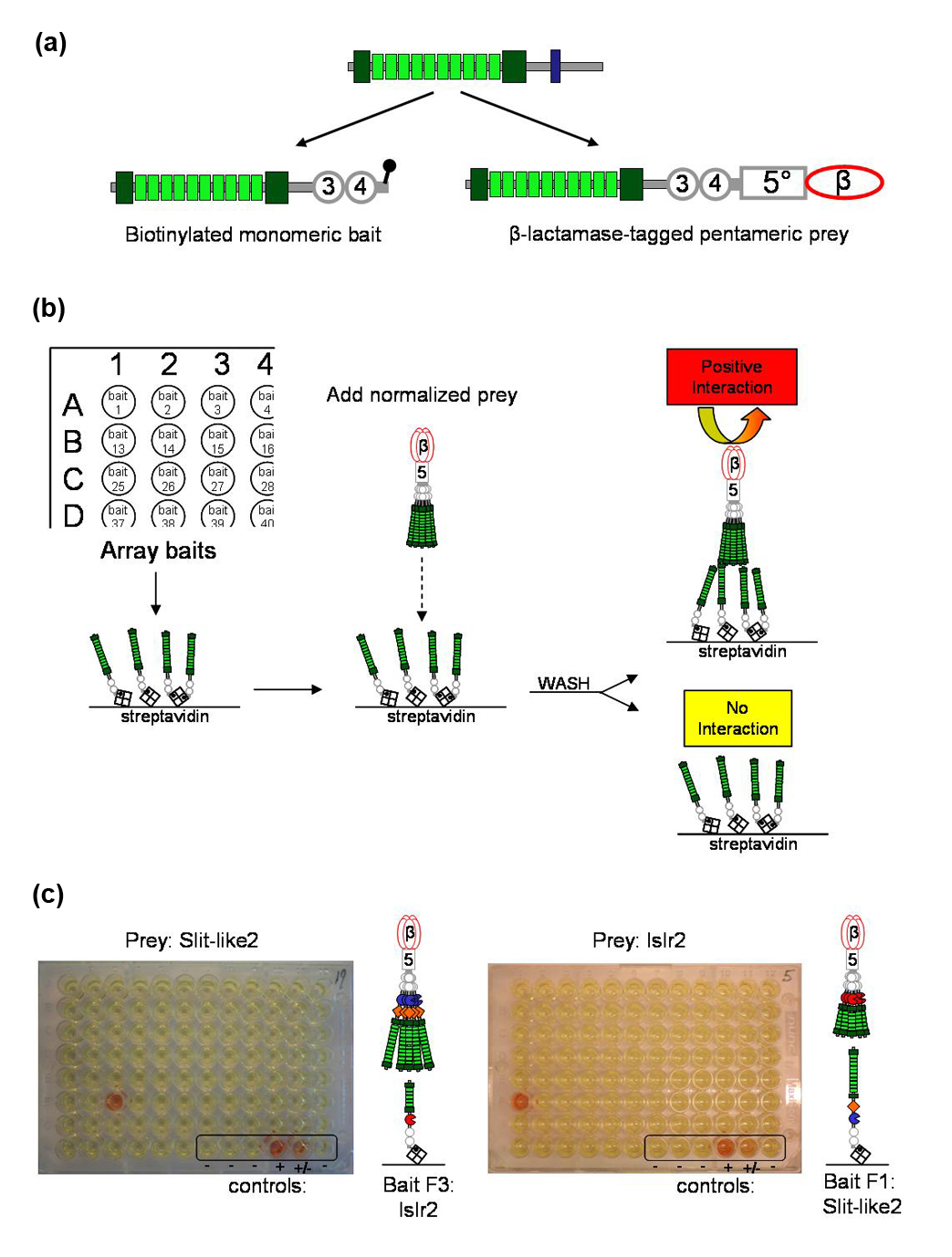

Supplement: Additional data file 1 — (a) The entire ectodomain of each LRR receptor was expressed in mammalian cells as both a bait and a prey. The bait proteins were biotinylated monomers, each containing a carboxy-terminal tag of the rat CD4 domains 3 and 4 and an enzymatically biotinylatable sequence. The prey also contained the rat CD4 tag but was followed by a pentamerization sequence derived from rat cartilage oligomeric matrix protein (5°) and the beta-lactamase enzyme. The expression levels of both bait and prey were measured and normalized. (b) The library of bait proteins was arrayed on a streptavidin-coated 96-well microtiter plate and a normalized prey protein added. After a brief wash, binding was determined by adding the colorimetric beta-lactamase substrate, nitrocefin: positive wells turned red. (c) Actual screening plates showing the Islr2/Slit-like2 interaction detected in a reciprocal fashion. The left panel shows Islr2 as the bait protein and Slit-like2 as the prey; the right panel shows the interaction in the reciprocal orientation. [file gb-2009-10-9-r99-S1.TIFF]

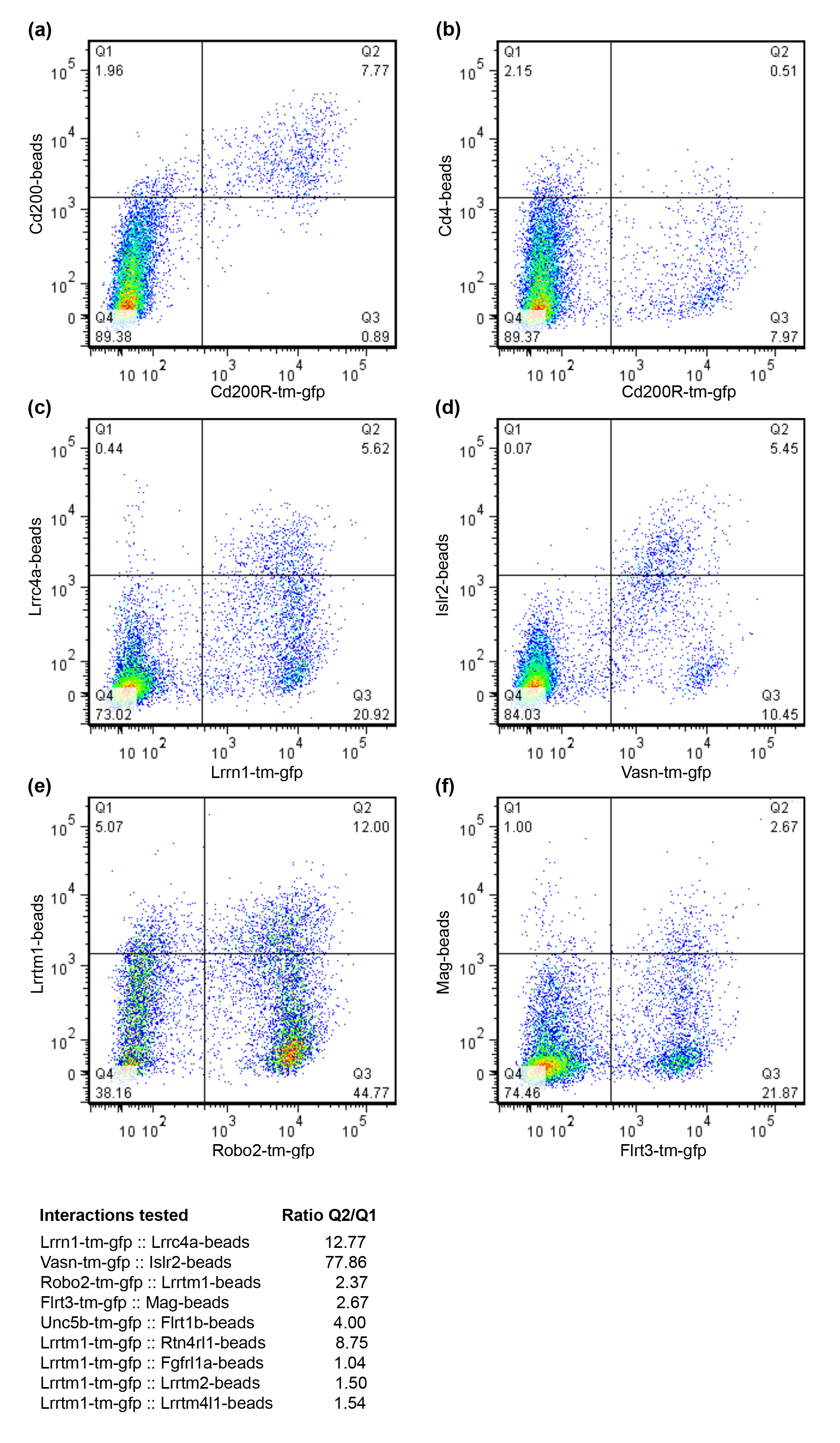

Supplement: Additional data file 2 — Interactions identified using AVEXIS were validated by immobilizing the biotinylated bait protein around a Nile Red fluorescent bead (shown on the y-axis) and presenting them to cells transfected with the extracellular regions of cell surface receptor proteins expressed as a TM-GFP chimera (x-axis). The percentage of events counted in each of the quadrants (Q1 to Q4) is shown. (a,b) Controls showing positive staining of rat Cd200-TM-GFP with rat Cd200R-coated beads (a) but not Cd4d3+4-coated beads (b). (c-f) Examples of interactions Lrrc4a-Lrrn1 (c), Islr2-Vasn (d), Lrrtm1-Robo2 (e) and Mag-Flrt3 (f) showing beads associating with transfected cells. Interactions were called as positive when beads preferentially associated with GFP-positive transfected cells (Q2:Q1 was greater than 1). [file gb-2009-10-9-r99-S2.TIFF]

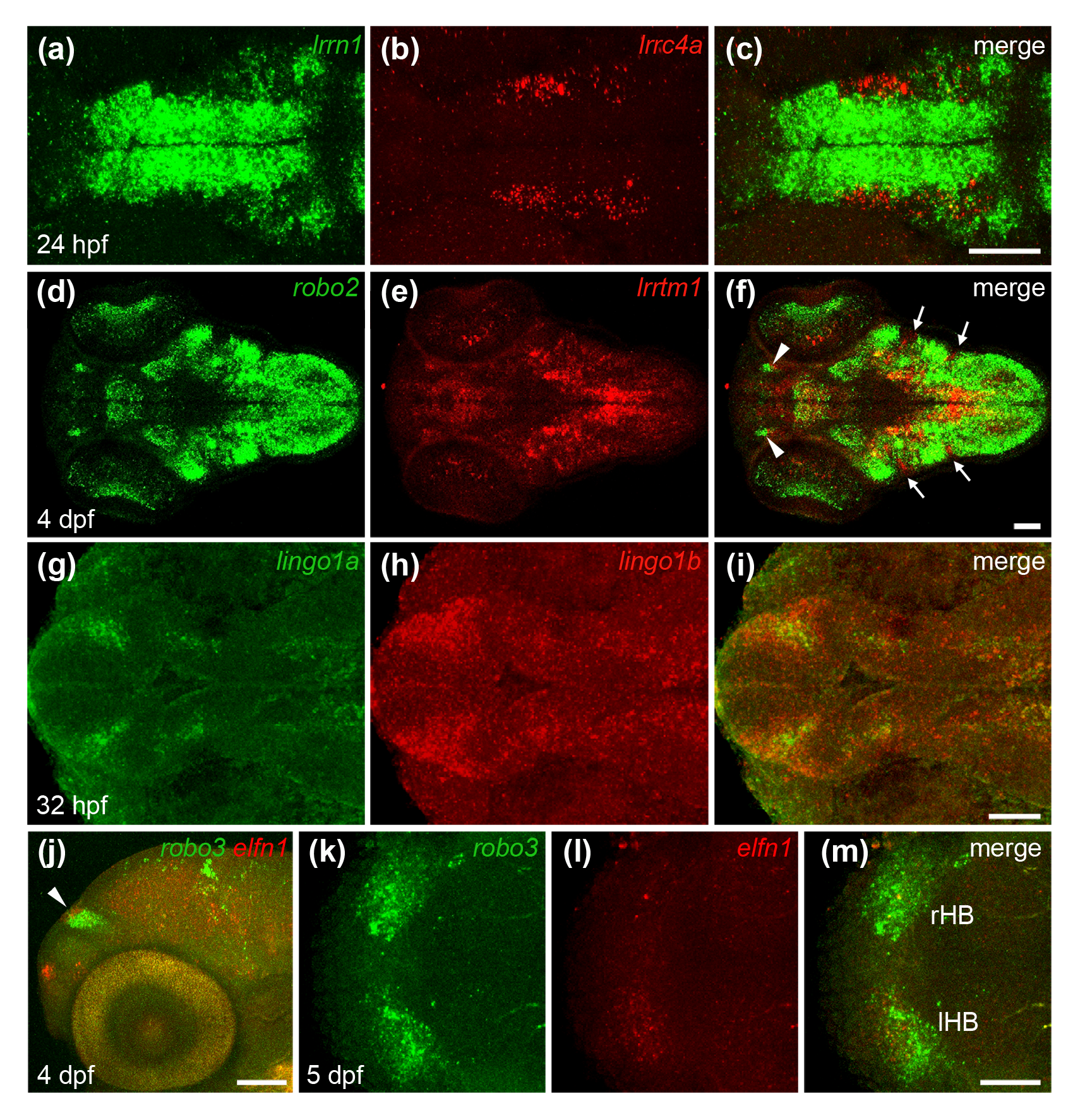

Supplement: Additional data file 3 — (a-c) Dorsal view of the zebrafish midbrain at 24 hours post-fertilization showing the nlrr1 gene (a) is expressed throughout the neuroepithelium of the midbrain, whereas its receptor, ngl2 (b), is restricted to two lateral midbrain domains that directly abut the nlrr1 expression domain (c). (d-f) A single optical section through the brain of a 4 days post-fertilization (dpf) zebrafish larva showing that robo2 (d) and lrrtm1 (e) are expressed in restricted patterns within all brain regions. The merge (f), shows largely non-overlapping, adjacent expression, particularly in a forebrain nucleus (arrowheads) and the hindbrain (arrows). (g-i) Dorsal view of the forebrain and partial midbrain of a 32 hours post-fertilization zebrafish larva showing that lingo1a (g) and lingo1b (h) are expressed in partially overlapping domains in the telencephalon (i). (j) Lateral view of a 4 dpf zebrafish larva showing robo3 expression in green and elfn1 expression in red. Both genes are expressed in the habenula nucleus in the dorsal forebrain (arrowhead). (k-m) Dorsal view of the forebrain region of a 5 dpf zebrafish larva showing that robo3 is expressed symmetrically in both habenula nuclei (k), whereas elfn1 (l,m) is expressed asymmetrically with higher expression levels in the left nucleus (lHB). All images are two-color wholemount in situ hybridizations with anterior left; cartoons depicting the interacting receptor-ligand pairs are shown in the left panels. Scale bar: 50 μm (a-c,d-f,g-j,k-m); 93 μm (j). [file gb-2009-10-9-r99-S3.TIFF]
