# Supplementary material for: A cell surface interaction network of neural leucine-rich repeat receptors
Source: Genome Biol. 2009 Sep 18;10(9):R99. doi: 10.1186/gb-2009-10-9-r99 (PMC2768988; doi:10.1186/gb-2009-10-9-r99)
Supplement: Additional data file 4 — Each gene is numbered according to its phylogenetic relationship as shown in Figure 1a and therefore clustered into LRR subfamilies as indicated. Listed for each gene are a systematic name with a cssl:d0 prefix, the current official ZFIN nomenclature, a proposed new nomenclature where appropriate (and used throughput this paper), GenBank accession number, the final carboxy-terminal amino acid of the ectodomain at which the truncation was made (the truncation site, 'Trunc.') and the closest human BLASTP match together with the percentage sequence identity. [file gb-2009-10-9-r99-S4.DOC]

**Additional data file 4.**

| **Number** | **Gene name** | **ZFIN name** | **Acc. No.** | **Trunc.** | **%ID** | **Closest human BLAST match** |
| --- | --- | --- | --- | --- | --- | --- |
|  |  |  |  |  |  |  |
|  | ***LINGOs*** |  |  |  |  |  |
| **1** | *cssl:d0317* | *lingo1b* | CU468744 | 553P | 395/551 (71%) | LRRN6A |
| **2** | *cssl:d0342* | *lingo1a* | CU468767 | 549T | 440/550 (80%) | LRRN6A |
| **3** | *cssl:d0348* | *sc:d0348* | CU468772 | 542S | 336/546 (61%) | LRRN6C |
| **4** | *cssl:d0353* | *si:dkey-172o19.1* | CU468775 | 539T | 311/513 (60%) | SIMILAR TO LRRN6C |
| **5** | *cssl:d0354* | *si:dkeyp-24a7.7* | CU468776 | 538T | 270/519 (52%) | LRRN6A |
|  |  |  |  |  |  |  |
|  | ***LRFNs*** |  |  |  |  |  |
| **6** | *cssl:d0343* | *sc:d0343* | CU468768 | 528G | 436/526 (82%) | LRFN-5 |
| **7** | *cssl:d0350* | *sc:d0350* | CU468774 | 538G | 380/528 (71%) | LRFN-5 |
| **8** | *cssl:d0381* | *zgc:63670* | CU468795 | 523G | 327/510 (64%) | LRFN-1 |
|  |  |  |  |  |  |  |
|  | ***LRRNs*** |  |  |  |  |  |
| **9** | *cssl:d0329* | *lrrn3* | CU468756 | 624G | 367/612 (59%) | LRRN3 |
| **10** | *cssl:d0349* | *lrrn1* | CU468773 | 631T | 503/631 (79%) | NLRR-1 |
| **11** | *cssl:d0413* | *sc:d0413* | CU468799 | 656P | 343/642 (53%) | NLRR-1 |
|  |  |  |  |  |  |  |
|  | ***LRRC4s*** |  |  |  |  |  |
| **12** | *cssl:d0316* | *sc:d0316* | CU468743 | 508G | 368/509 (72%) | NETRIN-G1 LIGAND |
| **13** | *cssl:d0565* | *lrrc4c* | CU468806 | 534T | 423/535 (79%) | NETRIN-G1 LIGAND |
| **14** | *cssl:d0383* | *sc:d0383* | CU468796 | 534K | 391/526 (74%) | LEUCINE-RICH REPEAT-CONTAINING PROTEIN 4B |
| **15** | *cssl:d0365* | *lrrc4a* | CU468781 | 543T | 381/549 (69%) | NAG14 |
|  |  |  |  |  |  |  |
| **16** | *cssl:d0332* | *si:ch211-145d10.6* | CU468759 | 245T | 118/223 (52%) | LEUCINE RICH REPEAT CONTAINING 38 |
|  |  |  |  |  |  |  |
|  | ***LRRC3s*** |  |  |  |  |  |
| **17** | *cssl:d0321* | *sc:d0321* | CU468748 | 194T | 79/165 (47%) | SIMILAR TO LEUCINE RICH REPEAT CONTAINING 3 |
| **18** | *cssl:d0330* | *sc:d0330* | CU468757 | 204R | 66/150 (44%) | HCG2044133 |
| **19** | *cssl:d0327* | *zgc:162270* | CU468754 | 201T | 121/206 (58%) | LRRC3B |
| **20** | *cssl:d0333* | *si:dkey-253a1.3* | CU468760 | 209T | 87/172 (50%) | LRRC3 |
|  |  |  |  |  |  |  |
| **21** | *cssl:d0337* | *lrrc24* | CU468764 | 444T | 220/419 (52%) | LRRC24 |
|  |  |  |  |  |  |  |
|  | ***FLRTs*** |  |  |  |  |  |
| **22** | *cssl:d0318* | *flrt1b* | CU468745 | 574P | 322/569 (56%) | FLRT1 |
| **23** | *cssl:d0372* | *flrt1a* | CU468788 | 558P | 332/552 (60%) | FLRT1 |
| **24** | *cssl:d0376* | *flrt3* | CU468792 | 527P | 374/528 (70%) | FLRT3 |
| **25** | *cssl:d0374* | *sc:d0374* | CU468790 | 541P | 323/548 (58%) | FLRT2 |
| **26** | *cssl:d0561* | *sc:d0374* | CU468804 | 252P | 156/221 (70%) | FLRT2 |
|  |  |  |  |  |  |  |
| **27** | *cssl:d0331* | *lrtm2* | CU468758 | 306R | 154/272 (56%) | LRR AND TRANSMEMBRANE DOMAIN-CONTAINING PROTEIN 2 PRECURSOR |
|  |  |  |  |  |  |  |
|  | ***RTN4Rs*** |  |  |  |  |  |
| **28** | *cssl:d0326* | *rtn4rl2b* | CU468753 | 432P | 192/376 (51%) | NgR2 |
| **29** | *cssl:d0564* | *rtn4rl2a* | CU468805 | 458G | 200/399 (50%) | NgR2 |
| **30** | *cssl:d0325* | *rtn4rl1* | CU468752 | 447P | 241/431 (55%) | RETICULON 4 RECEPTOR-LIKE 1 |
| **31** | *cssl:d0335* | *rtn4r* | CU468762 | 459S | 205/450 (45%) | NgR |
|  |  |  |  |  |  |  |
|  | ***LRRTMs*** |  |  |  |  |  |
| **32** | *cssl:d0320* | *lrrtm2* | CU468747 | 447R | 269/435 (61%) | LRRTM2 |
| **33** | *cssl:d0378* | *lrrtm1* | CU468794 | 429K | 266/433 (61%) | LRRTM1 |
| **34** | *cssl:d0340* | *lrrtm4l2* | CU468765 | 445K | 229/422 (54%) | LRRTM4 |
| **35** | *cssl:d0341* | *lrrtm4l1* | CU468766 | 456K | 233/386 (60%) | LRRTM4 |
|  |  |  |  |  |  |  |
|  | ***LRRC21/22*** |  |  |  |  |  |
| **36** | *cssl:d0312* | *zgc:109962* | CU468740 | 459R | 231/462 (50%) | LEUCINE RICH REPEAT CONTAINING 22 |
| **37** | *cssl:d0313* | *lrit1* | CU468741 | 546N | 271/546 (49%) | PAL |
|  |  |  |  |  |  |  |
| **38** | *cssl:d0373* | *islr2* | CU468789 | 567P | 254/597 (42%) | ISLR2 |
|  |  |  |  |  |  |  |
|  | ***ELFNs*** |  |  |  |  |  |
| **39** | *cssl:d0369* | *elfn1* | CU468785 | 420T | 221/376 (58%) | SIMLIAR TO SLIT HOMOLOG 1 |
| **40** | *cssl:d0398* | *elfn2* | CU468797 | 403T | 243/380 (63%) | LRFN-6 |
|  |  |  |  |  |  |  |
|  | ***AMIGOs*** |  |  |  |  |  |
| **41** | *cssl:d0322* | *si:ch211-77c7.1* | CU468749 | 368G | 188/341 (55%) | AMIGO |
|  |  |  |  |  |  |  |
| **42** | *cssl:d0336* | *vasn* | CU468763 | 583P | 242/562 (43%) | SLIT-LIKE 2 |
|  |  |  |  |  |  |  |
|  | ***5T4s*** |  |  |  |  |  |
| **43** | *cssl:d0323* | *sc:d0323* | CU468750 | 315P | 107/283 (37%) | 5T4 ONCOFETAL TROPHOBLAST GLYCOPROTEIN |
| **44** | *cssl:d0347* | *sc:d0347* | CU468771 | 316P | 105/283 (37%) | 5T4 ONCOFETAL TROPHOBLAST GLYCOPROTEIN |
| **45** | *cssl:d0324* | *tpbgl* | CU468751 | 307S | 110/297 (37%) | 5T4 ONCOFETAL TROPHOBLAST GLYCOPROTEIN |
|  |  |  |  |  |  |  |
| **46** | *cssl:d0328* | *zgc:100989* | CU468755 | 640S | 237/592 (40%) | LEUCINE RICH REPEAT CONTAINING 33 |
|  |  |  |  |  |  |  |
|  | ***TLRs*** |  |  |  |  |  |
| **47** | *cssl:d0554* | *tlr8b* | CU468802 | 802S | 331/820 (40%) | TOLL-LIKE RECEPTOR 8 |
| **48** | *cssl:d0334* | *tlr22* | CU468761 | 740N | 198/728 (27%) | TOLL-LIKE RECEPTOR 3 PRECURSOR |
|  |  |  |  |  |  |  |
|  | ***SLITRKs*** |  |  |  |  |  |
| **49** | *cssl:d0319* | *sc:d0319* | CU468746 | 622P | 458/624 (73%) | SLIT AND NTRK-LIKE FAMILY, MEMBER 2 |
| **50** | *cssl:d0344* | *none* | CU468769 | 619S | 461/625 (73%) | SLIT AND NTRK-LIKE FAMILY, MEMBER 4 |
|  |  |  |  |  |  |  |
| **51** | *cssl:d0557* | *lrrc15* | CU468803 | 522K | 175/439 (39%) | LRRC15 / LIB |
|  |  |  |  |  |  |  |
| **52** | *cssl:d0367* | *si:ch211-124k10.1* | CU468783 | 393L | 195/388 (50%) | LEUCINE-RICH REPEAT-CONTAINING G PROTEIN-COUPLED RECEPTOR 7 |
|  |  |  |  |  |  |  |
|  | ***TRKs*** |  |  |  |  |  |
| **53** | *cssl:d0314* | *si:ch211-220b11.1* | CU468742 | 401P | 160/375 (42%) | NTRK2 |
