# Supplementary material for: A cell surface interaction network of neural leucine-rich repeat receptors
Source: Genome Biol. 2009 Sep 18;10(9):R99. doi: 10.1186/gb-2009-10-9-r99 (PMC2768988; doi:10.1186/gb-2009-10-9-r99)
Supplement: Additional data file 5 — Each IgSF ectodomain is numbered in the order of its phylogenetic relationship and corresponds to the numbers in the LRR-IgSF binding grid (Figure 1c). Each gene is given a systematic identifier, the cssl:d prefix, which is listed together with the current official ZFIN nomenclature (note identical gene names indicate splice variants), GenBank accession number and the closest human BLASP match, together with the percentage sequence identity. Twenty-eight proteins indicated by asterisks were also produced as prey proteins and, therefore, screened in both bait-prey orientations. One protein, Sc:d805 (number 28 in the table), interacted with > 50% of the library as both a prey and a bait and was therefore excluded from subsequent analysis. [file gb-2009-10-9-r99-S5.DOC]

**Additional file 5.**

| **Number** | **Gene name** | **ZFIN name** | **Acc. No.** | **%ID** | **Closest human BLAST match** |
| --- | --- | --- | --- | --- | --- |
| **1** | *cssl:d834* | *zgc:165604* | CU458996 | 103/286 (36%) | IGSF11A |
| **2** | *cssl:d108* | *cxadr* | CU458753 | 159/363 (43%) | COXSACKIEVIRUS AND ADENOVIRUS RECEPTOR |
| **3** | *cssl:d144* | *sc:d0144* | CU458914 | 70/209 (33%) | A33 ANTIGEN |
| **4** | *cssl:d188* | *igsf21b* | CU458945 | 292/432 (67%) | IGSF21 |
| ***5** | *cssl:d809* | *nitr5* | CU458866 | 42/127 (33%) | TRA |
| **6** | *cssl:d203* | *vstm2l* | CU458955 | 84/156 (53%) | C20ORF102 |
| ***7** | *cssl:d807* | *si:dkey-63d15.12* | CU458864 | 35/115 (30%) | TRANSMEMBRANE AND IMMUNOGLOBULIN DOMAIN-CONTAINING PROTEIN 2 |
| **8** | *cssl:d228* | *bcam* | CU458967 | 31/101 (30%) | VEGA:OTTHUMP00000066243 |
| **9** | *cssl:d127* | *vcam1* | CU458903 | 131/468 (27%) | VASCULAR CELL ADHESION PROTEIN 1 |
| **10** | *cssl:d233* | *cntfr* | CU458971 | 180/322 (55%) | CNTFR |
| **11** | *cssl:d199* | *il11ra* | CU458952 | 97/282 (34%) | IL-11R |
| **12** | *cssl:d131* | *nlcam* | CU458904 | 186/549 (33%) | CD166 |
| **13** | *cssl:d176* | *nlcam* | CU458936 | 197/569 (34%) | CD166 |
| **14** | *cssl:d215* | *alcam* | CU458962 | 198/551 (35%) | CD166 |
| ***15** | *cssl:d822* | *alcam* | CU458879 | 189/506 (37%) | CD166 |
| ***16** | *cssl:d830* | *musk* | CU458887 | 243/462 (52%) | MUSK |
| ***17** | *cssl:d831* | *musk* | CU458888 | 75/151 (49%) | MUSK |
| **18** | *cssl:d173* | *sc:d173* | CU458933 | 48/209 (22%) | CEA |
| **19** | *cssl:d142* | *zgc:77222* | CU458912 | 228/605 (37%) | IGSF8 |
| ***20** | *cssl:d808* | *sc:d808* | CU458865 | 31/110 (28%) | MYOSIN LIGHT CHAIN KINASE |
| **21** | *cssl:d143* | *si:dkey-222f2.7* | CU458913 | 23/89 (25%) | CD2 |
| **22** | *cssl:d141* | *si:dkey-222f2.7* | CU458911 | 23/89 (25%) | CD2 |
| **23** | *cssl:d220* | *sc:d0220* | CU458966 | 45/192 (23%) | CD48 |
| **24** | *cssl:d217* | *sc:d217* | CU458964 | 55/212 (25%) | ENSEMBL:ENSP00000364178 |
| **25** | *cssl:d191* | *sc:d191* | CU458948 | 29/113 (25%) | CD48 |
| **26** | *cssl:d158* | *sc:d158* | CU458925 | 27/96 (28%) | V1-3 PROTEIN |
| **27** | *cssl:d114* | *kita* | CU458898 | 353/842 (41%) | MAST/STEM CELL GROWTH FACTOR RECEPTOR |
| ***28** | *cssl:d805* | *sc:d805* | CU458862 | 52/138 (37%) | C10ORF72 |
| **29** | *cssl:d116* | *csf1r* | CU458899 | 242/646 (37%) | MACROPHAGE COLONY STIMULATING FACTOR 1 RECEPTOR |
| **30** | *cssl:d172* | *zgc:162565* | CU458932 | 149/270 (55%) | NEUROPLASTIN |
| **31** | *cssl:d229* | *bsg* | CU458968 | 103/267 (38%) | BASINGIN |
| **32** | *cssl:d169* | *sc:d169* | CU458931 | 149/256 (58%) | BASINGIN |
| ***33** | *cssl:d810* | *sc:d810* | CU458867 | 56/181 (30%) | TRANSMEMBRANE AND IMMUNOGLOBULIN DOMAIN-CONTAINING PROTEIN 1 |
| ***34** | *cssl:d832* | *unc5b* | CU458889 | 252/337 (74%) | UNC5B |
| ***35** | *cssl:d819* | *fgfrl1b* | CU458876 | 221/340 (65%) | FGFRL |
| **36** | *cssl:d155* | *fgfrl1a* | CU458922 | 298/464 (64%) | FGFRL1 |
| **37** | *cssl:d109* | *fgfr4* | CU458754 | 210/367 (57%) | FGFR4 |
| **38** | *cssl:d107* | *fgfr1a* | CU458752 | 274/483 (56%) | FGFR1 |
| ***39** | *cssl:d811* | *fgfr1b* | CU458868 | 175/253 (69%) | FGFR1 |
| ***40** | *cssl:d821* | *mag* | CU458878 | 210/491 (42%) | MAG |
| **41** | *cssl:d190* | *sc:d190* | CU458947 | 47/169 (27%) | SIALOADHESIN |
| **42** | *cssl:d205* | *sc:d0205* | CU458816 | 464/712 (65%) | NEUROFASCIN |
| ***43** | *cssl:d824* | *nadl1.1* | CU458881 | 413/1112 (37%) | L1 |
| ***44** | *cssl:d823* | *sc:d0823* | CU458880 | 146/292 (50%) | ROBO1 |
| **45** | *cssl:d100* | *robo1* | CU458748 | 646/871 (74%) | ROBO1 |
| **46** | *cssl:d102* | *robo3* | CU458749 | 590/915 (64%) | ROBO2 |
| ***47** | *cssl:d829* | *robo2* | CU458886 | 693/857 (80%) | ROBO2 |
| **48** | *cssl:d104* | *boc* | CU458750 | 251/420 (59%) | VEGA:OTTHUMP00000096002 |
| **49** | *cssl:d202* | *sc:d0202* | CU458954 | 58/279 (20%) | VEGA:OTTHUMP00000012652 |
| **50** | *cssl:d139* | *sc:d0139* | CU458910 | 59/279 (21%) | VEGA:OTTHUMP00000038305 |
| **51** | *cssl:d218* | *cadm2a* | CU458965 | 264/410 (64%) | IGSF4D |
| ***52** | *cssl:d820* | *kirrel* | CU458877 | 330/487 (67%) | KIRRE |
| ***53** | *cssl:d814* | *negr1* | CU458871 | 191/316 (60%) | NEURONAL GROWTH REGULATOR 1 |
| **54** | *cssl:d168* | *zgc:110372* | CU458930 | 173/307 (56%) | SIM TO OBCAM |
| ***55** | *cssl:d815* | *ntm* | CU458872 | 159/307 (51%) | OBCAM |
| ***56** | *cssl:d827* | *ncam2* | CU458884 | 425/691 (61%) | NCAM2 |
| **57** | *cssl:d097* | *ncam3* | CU458747 | 471/797 (59%) | NCAM |
| ***58** | *cssl:d825* | *ncam3* | CU458882 | 430/718 (59%) | NCAM |
| ***59** | *cssl:d828* | *ncam1* | CU458885 | 441/712 (61%) | NCAM |
| **60** | *cssl:d206* | *pvrl3l* | CU458957 | 195/469 (41%) | POLIOVIRUS RECEPTOR-RELATED 3 |
| **61** | *cssl:d124* | *pvrl3l* | CU458902 | 191/459 (41%) | POLIOVIRUS RECEPTOR-RELATED 3 |
| **62** | *cssl:d156* | *sc:d156* | CU458923 | 44/174 (25%) | CD22 |
| **63** | *cssl:d153* | *si:dkey-24p1.1* | CU458921 | 161/609 (26%) | CD22 |
| **64** | *cssl:d148* | *sc:d148* | CU458918 | 179/418 (42%) | SINGLE IG IL-1 RELATED RECEPTOR |
| **65** | *cssl:d136* | *sc:d136* | CU458907 | 61/242 (25%) | MAG-B |
| **66** | *cssl:d230* | *neo1* | CU458829 | 753/1092 (68%) | NEOGENIN |
| **67** | *cssl:d147* | *si:ch211-264f5.2* | CU458917 | 51/185 (27%) | POLY IG RECEPTOR |
| **68** | *cssl:d132* | *si:ch211-261c8.5* | CU458905 | 100/258 (38%) | EMBIGIN |
| **69** | *cssl:d185* | *sc:d185* | CU458942 | 45/125 (36%) | T-CELL IMMUNOGLOBULIN AND MUCIN DOMAIN-CONTAINING PROTEIN 4 |
| **70** | *cssl:d836* | *kitb* | CU458998 | 61/208 (29%) | MAST/STEM CELL GROWTH FACTOR RECEPTOR |
| **71** | *cssl:d111* | *dtk* | CU458897 | 345/675 (51%) | TYROSINE-PROTEIN KINASE RECEPTOR TYR03 |
| ***72** | *cssl:d816* | *cssl:d816* | CU458873 | 49/186 (26%) | CD200 |
| **73** | *cssl:d189* | *sc:d189* | CU458946 | 54/212 (25%) | CD200 |
| ***74** | *cssl:d817* | *sc:d0817* | CU458874 | 61/213 (28%) | CEA |
| **75** | *cssl:d175* | *sc:d0175* | CU458935 | 63/213 (29%) | CEA1 |
| **76** | *cssl:d179* | *si:dkey-11f4.16* | CU458939 | 60/209 (28%) | LFA3 |
| **77** | *cssl:d178* | *jam3* | CU458938 | 153/280 (54%) | JAM3 |
| **78** | *cssl:d216* | *f11r* | CU458963 | 105/268 (39%) | JAM-A |
| **79** | *cssl:d121* | *f11r* | CU458900 | 105/268 (39%) | JAM-A |
| **80** | *cssl:d187* | *jam2* | CU458944 | 104/233 (44%) | VEGA:OTTHUMP00000096102 |
| ***81** | *cssl:d813* | *si:ch211-160b11.5* | CU458870 | 78/199 (39%) | VEGA:OTTHUMP00000096102 |
| **82** | *cssl:d835* | *sc:d0835* | CU458997 | 78/248 (31%) | BUTYROPHILIN |
| ***83** | *cssl:d818* | *sc:d818* | CU458875 | 68/203 (33%) | TAPASIN |
| **84** | *cssl:d105* | *tpsn* | CU458751 | 68/215 (31%) | TAPASIN |
| **85** | *cssl:d163* | *zgc:158279* | CU458928 | 124/253 (49%) | VEGA:OTTHUMP00000175951 |
| ***86** | *cssl:d826* | *cntn2* | CU458883 | 271/498 (54%) | CONTACTIN-2 |
| **87** | *cssl:d232* | *wu:fu71h07* | CU458970 | 34/118 (28%) | MOG |
| **88** | *cssl:d183* | *si:dkey-222p3.1* | CU458941 | 36/124 (29%) | V-SET DOMAIN CONTAINING T CELL ACTIVATION INHIBITOR 1 |
| **89** | *cssl:d135* | *mpzl3* | CU458906 | 52/115 (45%) | VEGA:OTTHUMP00000165009 |
| **90** | *cssl:d186* | *sc:d0186* | CU458943 | 70/127 (55%) | P0 |
| ***91** | *cssl:d806* | *mpz* | CU458863 | 71/128 (55%) | P0 |
| **92** | *cssl:d174* | *mpzl2* | CU458934 | 65/139 (46%) | EVA1 |
| **93** | *cssl:d204* | *mpzl2* | CU458956 | 58/128 (45%) | EVA1 |
| **94** | *cssl:d166* | *zgc:91849* | CU458929 | 257/788 (32%) | IGSF2 |
| **95** | *cssl:d213* | *si:ch211-149p10.2* | CU458960 | 35/110 (31%) | BUTYROPHILIN |
| **96** | *cssl:d180* | *si:dkey-15j16.3* | CU458940 | 37/108 (34%) | A33 ANTIGEN |
| ***97** | *cssl:d812* | *sc:d812* | CU458869 | 166/240 (69%) | IGSF11 |
