# Supplementary material for: A cell surface interaction network of neural leucine-rich repeat receptors
Source: Genome Biol. 2009 Sep 18;10(9):R99. doi: 10.1186/gb-2009-10-9-r99 (PMC2768988; doi:10.1186/gb-2009-10-9-r99)
Supplement: Additional data file 6 — Interactions were classified into 12 groups (A to L) according to their behavior in the interaction screen using AVEXIS as taken from [24]; according to this scheme, no class B interactions were categorized. Interactions were considered as high confidence if they were positive in the primary screen and could be detected in both bait-prey orientations in either the primary or validation screens (classes A, C, D, E, F). IntAct accession numbers for both bait-prey orientations are provided where applicable. [file gb-2009-10-9-r99-S6.DOC]

**Additional file 6.**

**AVEXIS classifications:**

A: Homophilic interaction seen in both the primary and validation screens.

C: Heterophilic interaction positive in both bait:prey orientations in the primary and validation screens. Positive 4 out of 4 times tested.

D: Heterophilic interaction positive in both bait:prey orientations in either the primary or validation screens. Positive 3 out of 4 times tested.

E: Heterophilic interaction positive in one bait:prey orientation in the primary screen but other orientation not tested; positive in both orientations in the validation screen. Positive 3 out of 3 times tested.

F: Heterophilic interaction positive in one bait:prey orientation in the primary screen but other orientation not tested; also positive in one orientation only in validation screen (both tested) but in different orientation to the primary screen. Positive 2 out of 3 times tested.

G: Heterophilic interaction positive in one bait:prey orientation only in both the primary and validation screen. Positive 2 out of 4 times tested.

H: Heterophilic interaction negative in both bait:prey orientations in the primary screen but positive in both orientations in the validation screen. Positive 2 out of 4 times tested.

I: Heterophilic interaction positive in one bait:prey orientation in the primary screen but other orientation not tested; positive in only the same orientation in the validation sceen (both tested). Positive 2 out of 3 times tested.

J: Heterophilic interaction negative in the one tested bait:prey orientation in the primary screen but positive in both orientations in the validation screen. Positive 2 out of 3 times tested.

K: Heterophilic interaction positive in both bait:prey orientations in the primary screen but not seen in the validation screens. Positive 2 out of 4 times tested.

L: Heterophilic interaction positive in one bait:prey orientation in the primary screen; positive in the other orientation in the validation screen. Positive 2 out of 4 times tested.

Note that in AVEXIS classes G and I, the interaction is bait:prey orientation dependent. In these cases, the bait is listed as protein 1 and the prey as protein 2

| **Interaction number** | **Interaction** | | **AVEXIS**  **Class** | **IntAct accession number** | |
| --- | --- | --- | --- | --- | --- |
|  | **Protein 1** | **Protein 2** |  | **Bait (1)-prey (2)** | **Bait(2)-prey(1)** |
| ***High confidence*** |  |  |  |  |  |
| Homophilic |  |  |  |  |  |
| 1 | Lrrtm2 |  | A | EBI-2263346 | N/A |
| 2 | Lrrtm1 |  | A | EBI-2263359 | N/A |
| 3 | Flrt1a |  | A | EBI-2263370 | N/A |
| 4 | Lrrtm4l1 |  | A | EBI-2263381 | N/A |
| 5 | Lrrtm4l2 |  | A | EBI-2263394 | N/A |
| Heterophilic |  |  |  |  |  |
| 6 | Lrrn1 | Lrrc4c | C | EBI-2263409 | EBI-2263557 |
| 7 | Unc5b | Flrt1b | C | EBI-2263420 | EBI-2263570 |
| 8 | Unc5b | Flrt1a | C | EBI-2263433 | EBI-2263579 |
| 9 | Islr2 | Vasn | C | EBI-2263446 | EBI-2263588 |
| 10 | Lrrtm2 | Lrrtm1 | C | EBI-2263455 | EBI-2263599 |
| 11 | Lrrtm1 | Lrrtm4l1 | C | EBI-2263464 | EBI-2263608 |
| 12 | Lrrtm1 | Lrrtm4l2 | C | EBI-2263473 | EBI-2263617 |
| 13 | Unc5b | Flrt3 | D | EBI-2263484 | EBI-2263628 |
| 14 | Unc5b | Islr2 | D | EBI-2263493 | EBI-2263639 |
| 15 | Lrrtm1 | Fgfrl1b | D | EBI-2263504 | EBI-2263648 |
| 16 | Lrrtm4l1 | Lrrtm4l2 | D | EBI-2263513 | EBI-2263659 |
| 17 | Elfn2 | IgSF21 | E | EBI-2263522 | EBI-2263670 |
| 18 | Lrrtm1 | Boc | E | EBI-2263537 | EBI-2263679 |
| 19 | Lrrtm1 | Fgfrl1a | E | EBI-2263548 | EBI-2263692 |
| 20 | Elfn1 | Robo3 | F | EBI-2263725 | EBI-2263701 |
| 21 | Lrrtm4l1 | Boc | F | EBI-2263734 | EBI-2263714 |
| ***Lower confidence*** |  |  |  |  |  |
| Heterophilic |  |  |  |  |  |
| 22 | Lingo1a | Lingo1b | G | EBI-2263745 | N/A |
| 23 | Lrrn1 | Lrrc4a | G | EBI-2263758 | N/A |
| 24 | Lrrtm2 | Lrrtm4l2 | G | EBI-2263767 | N/A |
| 25 | Lrrc24 | Robo2 | G | EBI-2263776 | N/A |
| 26 | Lrrtm1 | Rtn4rl1 | H | EBI-2263789 | EBI-2263880 |
| 27 | Lrrtm4l1 | Lrrc24 | H | EBI-2263800 | EBI-2263889 |
| 28 | Flrt3 | Mag | H | EBI-2263811 | EBI-2263898 |
| 29 | Elfn2 | Boc | I | EBI-2263820 | N/A |
| 30 | Flrt3 | Boc | I | EBI-2263829 | N/A |
| 31 | Lrrtm1 | Fgfr4 | J | EBI-2263840 | EBI-2263909 |
| 32 | Robo2 | Lrrtm1 | K | EBI-2263851 | EBI-2263920 |
| 33 | Lrrtm2 | Lrrtm4l1 | K | EBI-2263860 | EBI-2263929 |
| 34 | Lrrtm1 | Mag | L | EBI-2263869 | EBI-2263938 |
